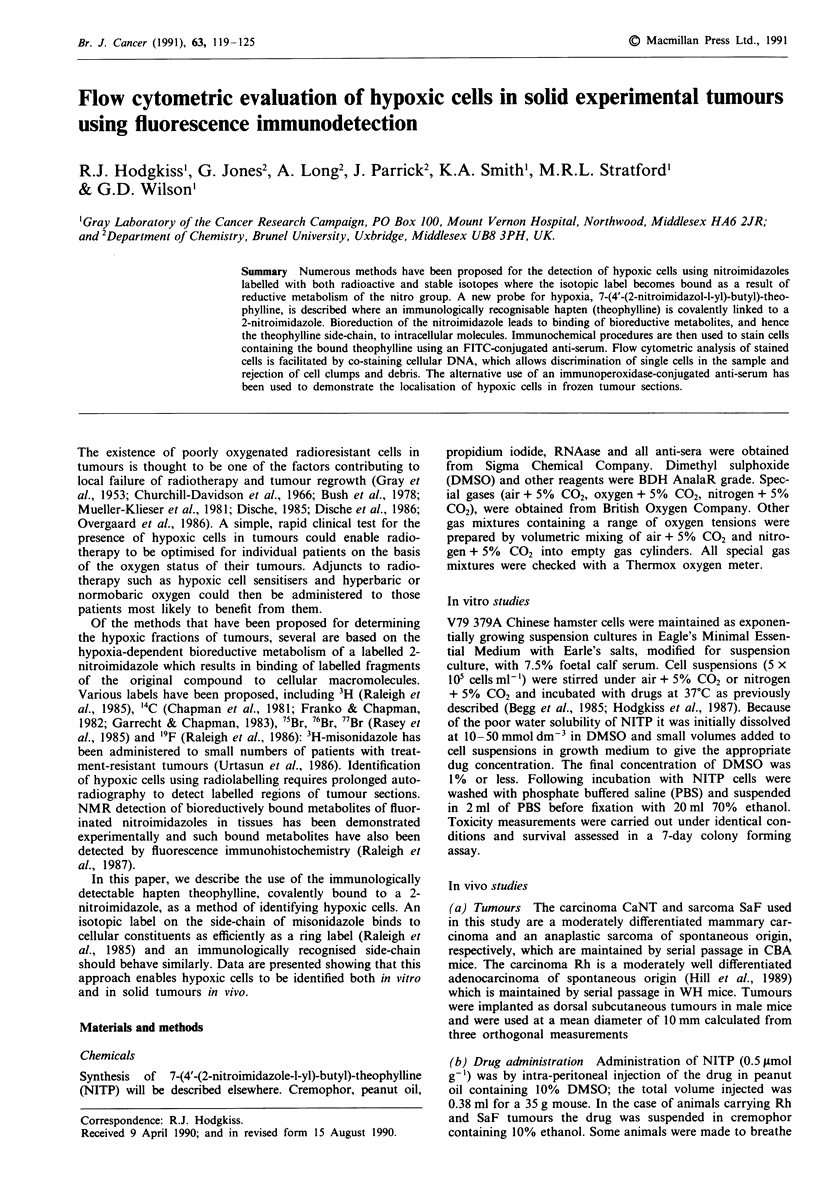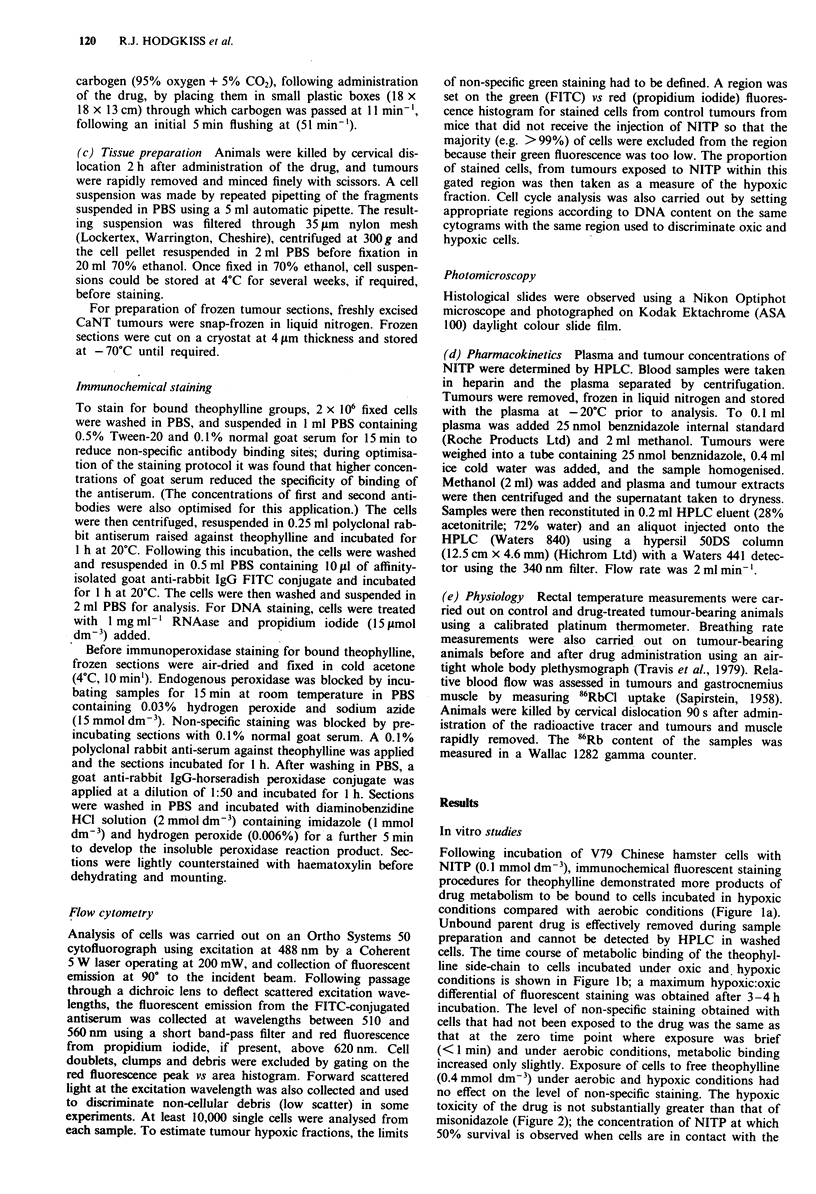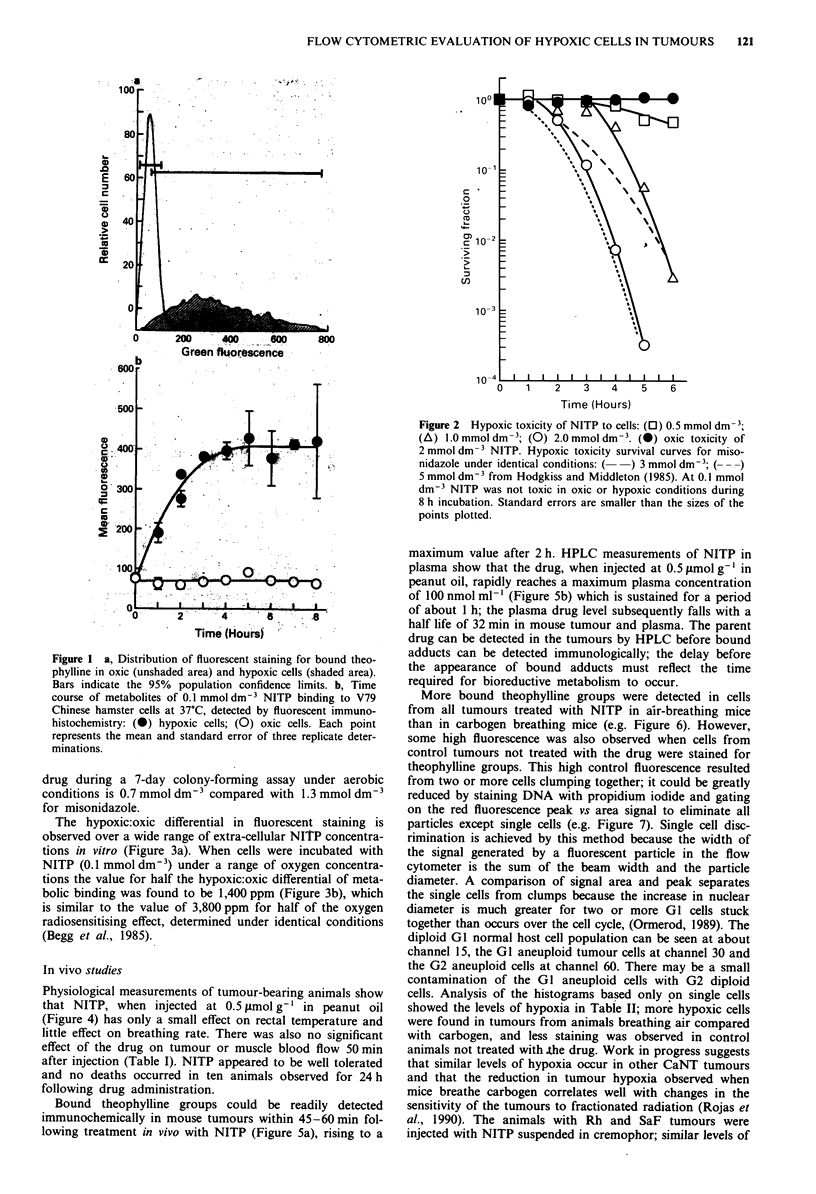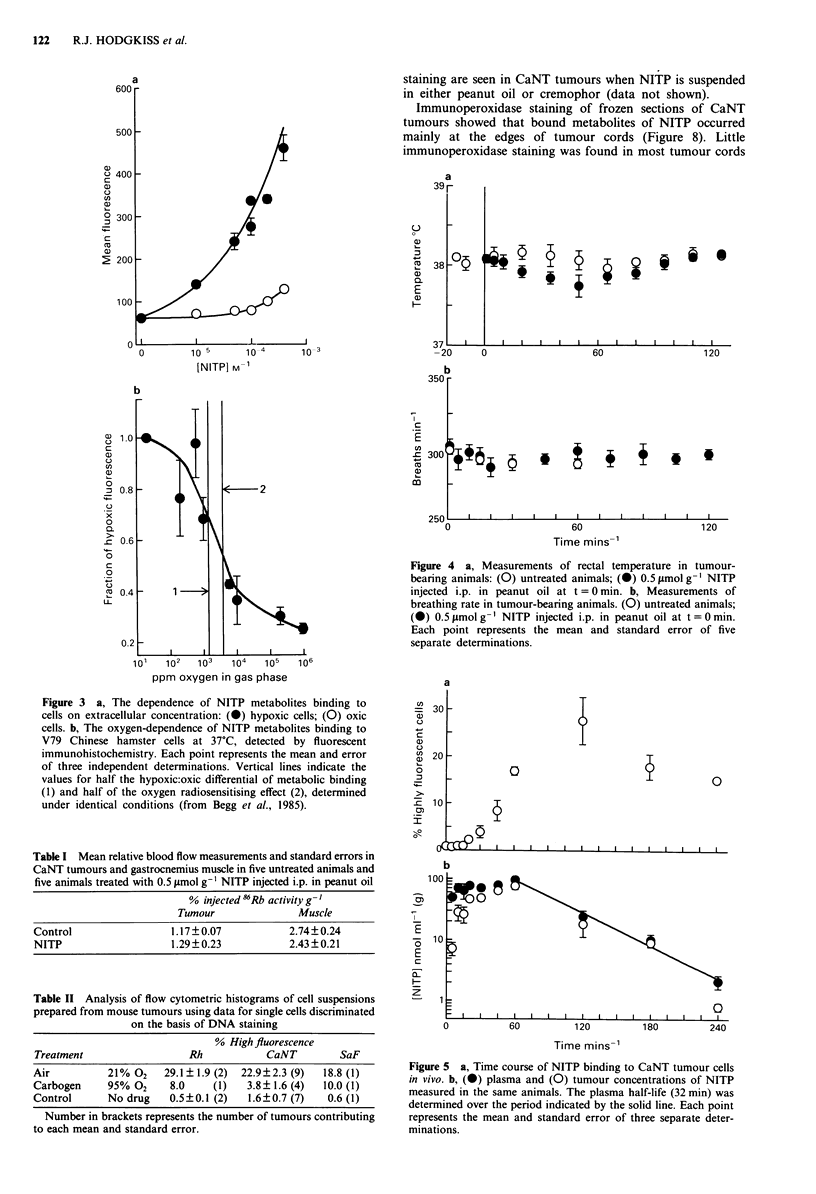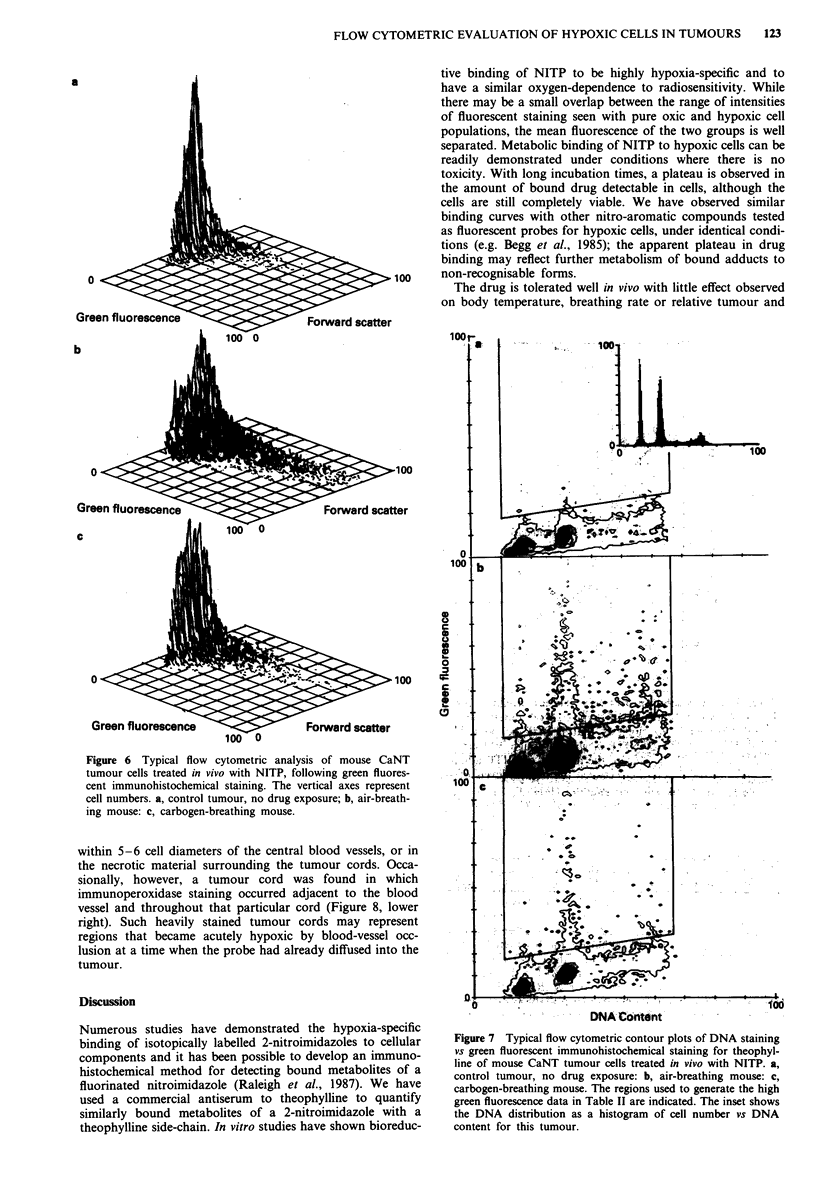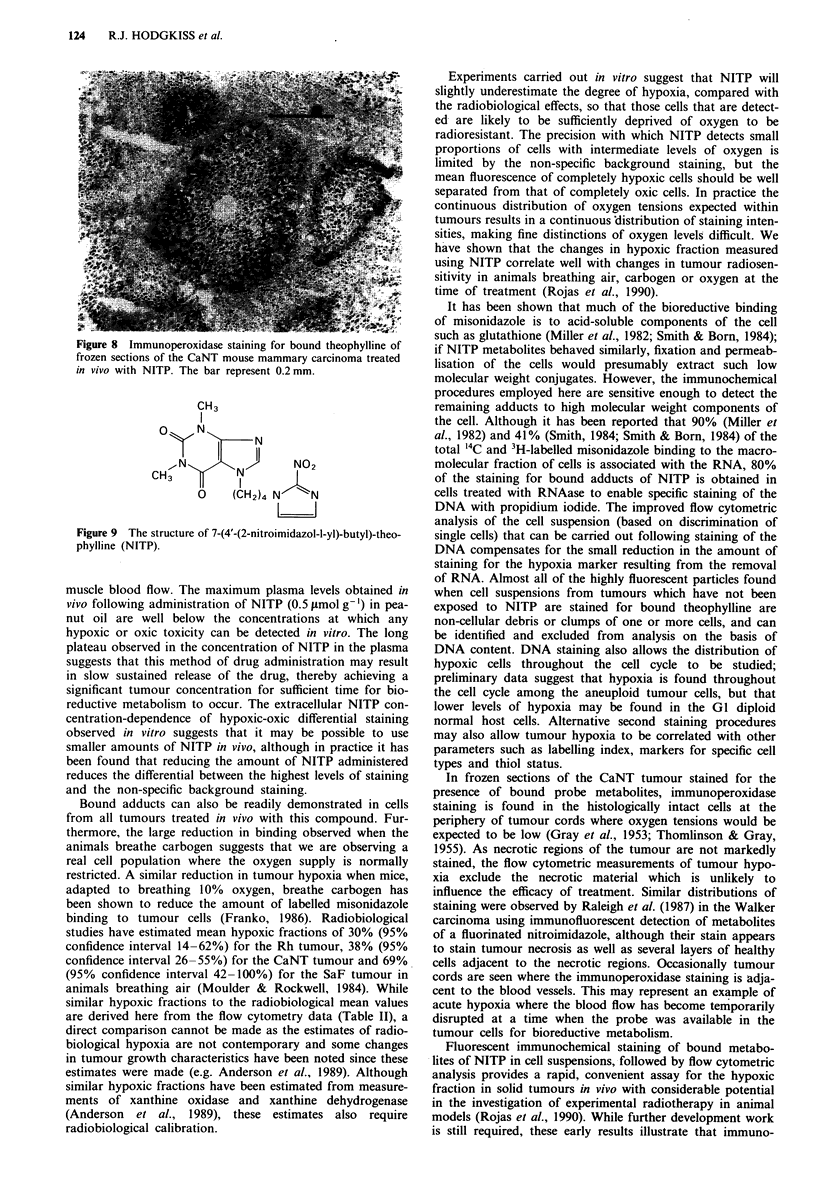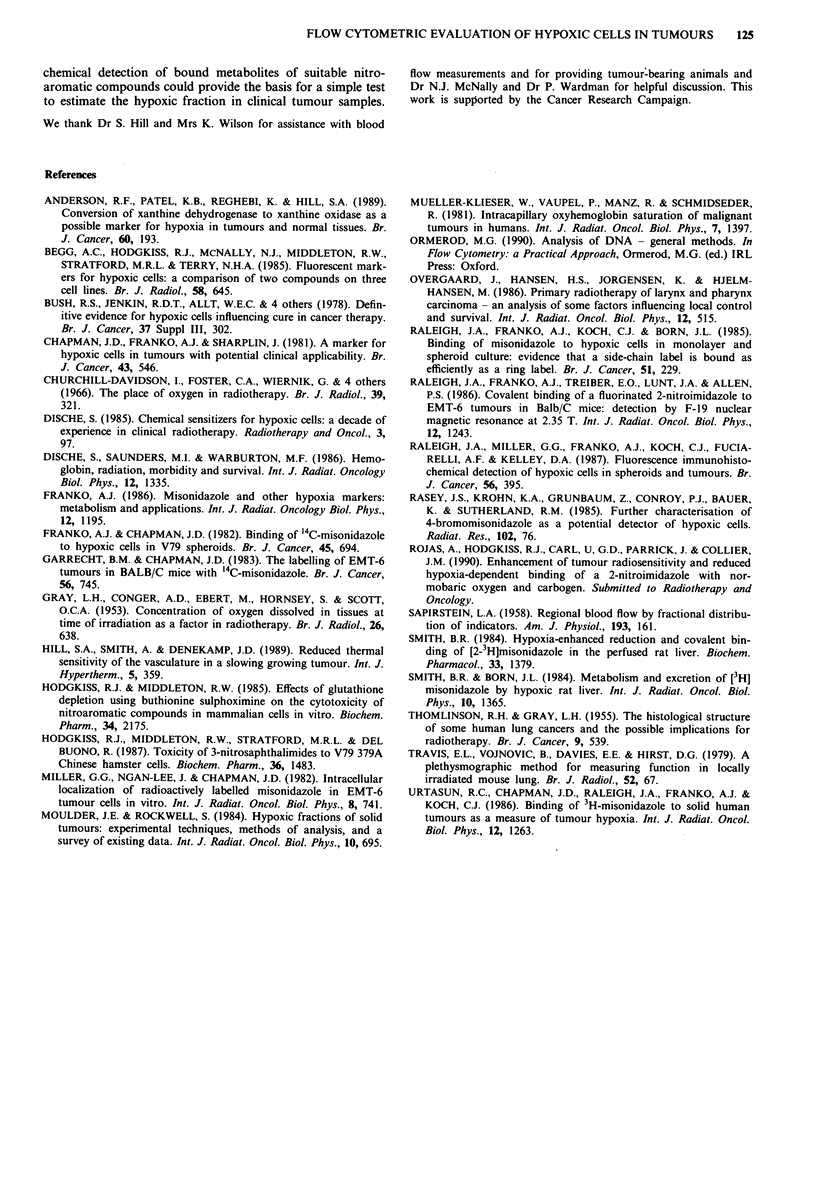# Flow cytometric evaluation of hypoxic cells in solid experimental tumours using fluorescence immunodetection.

**DOI:** 10.1038/bjc.1991.24

**Published:** 1991-01

**Authors:** R. J. Hodgkiss, G. Jones, A. Long, J. Parrick, K. A. Smith, M. R. Stratford, G. D. Wilson

**Affiliations:** Gray Laboratory of the Cancer Research Campaign, Mount Vernon Hospital, Northwood, Middlesex, UK.

## Abstract

**Images:**


Br. J. Cancer (1991), 63, 119-125 © Macmillan Press Ltd., 1991

### Flow cytometric evaluation of hypoxic cells in solid experimental tumours using fluorescence immunodetection

R.J. Hodgkiss[1], G. Jones[2], A. Long[2], J. Parrick[2], K.A. Smith[1], M.R.L. Stratford[1] & G.D. Wilson[1]

[1]*Gray Laboratory of the Cancer Research Campaign, PO Box 100, Mount Vernon Hospital, Northwood, Middlesex HA6 2JR; and* [2]*Department of Chemistry, Brunel University, Uxbridge, Middlesex UB8 3PH, UK.*

**Summary** Numerous methods have been proposed for the detection of hypoxic cells using nitroimidazoles labelled with both radioactive and stable isotopes where the isotopic label becomes bound as a result of reductive metabolism of the nitro group. A new probe for hypoxia, 7-(4'-(2-nitroimidazol-l-yl)-butyl)-theophylline, is described where an immunologically recognisable hapten (theophylline) is covalently linked to a 2-nitroimidazole. Bioreduction of the nitroimidazole leads to binding of bioreductive metabolites, and hence the theophylline side-chain, to intracellular molecules. Immunochemical procedures are then used to stain cells containing the bound theophylline using an FITC-conjugated anti-serum. Flow cytometric analysis of stained cells is facilitated by co-staining cellular DNA, which allows discrimination of single cells in the sample and rejection of cell clumps and debris. The alternative use of an immunoperoxidase-conjugated anti-serum has been used to demonstrate the localisation of hypoxic cells in frozen tumour sections.

The existence of poorly oxygenated radioresistant cells in tumours is thought to be one of the factors contributing to local failure of radiotherapy and tumour regrowth (Gray *et al.*, 1953; Churchill-Davidson *et al.*, 1966; Bush *et al.*, 1978; Mueller-Klieser *et al.*, 1981; Dische, 1985; Dische *et al.*, 1986; Overgaard *et al.*, 1986). A simple, rapid clinical test for the presence of hypoxic cells in tumours could enable radiotherapy to be optimised for individual patients on the basis of the oxygen status of their tumours. Adjuncts to radiotherapy such as hypoxic cell sensitisers and hyperbaric or normobaric oxygen could then be administered to those patients most likely to benefit from them.

Of the methods that have been proposed for determining the hypoxic fractions of tumours, several are based on the hypoxia-dependent bioreductive metabolism of a labelled 2-nitroimidazole which results in binding of labelled fragments of the original compound to cellular macromolecules. Various labels have been proposed, including $^{3}H$ (Raleigh *et al.*, 1985), $^{14}C$ (Chapman *et al.*, 1981; Franko & Chapman, 1982; Garrecht & Chapman, 1983), $^{75}Br$, $^{76}Br$, $^{77}Br$ (Rasey *et al.*, 1985) and $^{19}F$ (Raleigh *et al.*, 1986): $^{3}H$-misonidazole has been administered to small numbers of patients with treatment-resistant tumours (Urtasun *et al.*, 1986). Identification of hypoxic cells using radiolabelling requires prolonged autoradiography to detect labelled regions of tumour sections. NMR detection of bioreductively bound metabolites of fluorinated nitroimidazoles in tissues has been demonstrated experimentally and such bound metabolites have also been detected by fluorescence immunohistochemistry (Raleigh *et al.*, 1987).

In this paper, we describe the use of the immunologically detectable hapten theophylline, covalently bound to a 2-nitroimidazole, as a method of identifying hypoxic cells. An isotopic label on the side-chain of misonidazole binds to cellular constituents as efficiently as a ring label (Raleigh *et al.*, 1985) and an immunologically recognised side-chain should behave similarly. Data are presented showing that this approach enables hypoxic cells to be identified both *in vitro* and in solid tumours *in vivo*.

### Materials and methods

### Chemicals

Synthesis of 7-(4'-(2-nitroimidazole-l-yl)-butyl)-theophylline (NITP) will be described elsewhere. Cremophor, peanut oil, propidium iodide, RNAase and all anti-sera were obtained from Sigma Chemical Company. Dimethyl sulphoxide (DMSO) and other reagents were BDH AnalaR grade. Special gases (air + 5% $CO_2$, oxygen + 5% $CO_2$, nitrogen + 5% $CO_2$), were obtained from British Oxygen Company. Other gas mixtures containing a range of oxygen tensions were prepared by volumetric mixing of air + 5% $CO_2$ and nitrogen + 5% $CO_2$ into empty gas cylinders. All special gas mixtures were checked with a Thermox oxygen meter.

### In vitro *studies*

V79 379A Chinese hamster cells were maintained as exponentially growing suspension cultures in Eagle's Minimal Essential Medium with Earle's salts, modified for suspension culture, with 7.5% foetal calf serum. Cell suspensions ($5 \times 10^5$ cells $ml^{-1}$) were stirred under air + 5% $CO_2$ or nitrogen + 5% $CO_2$ and incubated with drugs at 37°C as previously described (Begg *et al.*, 1985; Hodgkiss *et al.*, 1987). Because of the poor water solubility of NITP it was initially dissolved at 10-50 mmol $dm^{-3}$ in DMSO and small volumes added to cell suspensions in growth medium to give the appropriate dug concentration. The final concentration of DMSO was 1% or less. Following incubation with NITP cells were washed with phosphate buffered saline (PBS) and suspended in 2 ml of PBS before fixation with 20 ml 70% ethanol. Toxicity measurements were carried out under identical conditions and survival assessed in a 7-day colony forming assay.

### In vivo *studies*

*(a) Tumours* The carcinoma CaNT and sarcoma SaF used in this study are a moderately differentiated mammary carcinoma and an anaplastic sarcoma of spontaneous origin, respectively, which are maintained by serial passage in CBA mice. The carcinoma Rh is a moderately well differentiated adenocarcinoma of spontaneous origin (Hill *et al.*, 1989) which is maintained by serial passage in WH mice. Tumours were implanted as dorsal subcutaneous tumours in male mice and were used at a mean diameter of 10 mm calculated from three orthogonal measurements

*(b) Drug administration* Administration of NITP (0.5 μmol $g^{-1}$) was by intra-peritoneal injection of the drug in peanut oil containing 10% DMSO; the total volume injected was 0.38 ml for a 35 g mouse. In the case of animals carrying Rh and SaF tumours the drug was suspended in cremophor containing 10% ethanol. Some animals were made to breathe

Correspondence: R.J. Hodgkiss.
Received 9 April 1990; and in revised form 15 August 1990.

carbogen (95% oxygen + 5% $CO_2$), following administration of the drug, by placing them in small plastic boxes (18 × 18 × 13 cm) through which carbogen was passed at 1 l min$^{-1}$, following an initial 5 min flushing at (5 l min$^{-1}$).

*(c) Tissue preparation* Animals were killed by cervical dislocation 2 h after administration of the drug, and tumours were rapidly removed and minced finely with scissors. A cell suspension was made by repeated pipetting of the fragments suspended in PBS using a 5 ml automatic pipette. The resulting suspension was filtered through 35 μm nylon mesh (Lockertex, Warrington, Cheshire), centrifuged at 300 *g* and the cell pellet resuspended in 2 ml PBS before fixation in 20 ml 70% ethanol. Once fixed in 70% ethanol, cell suspensions could be stored at 4°C for several weeks, if required, before staining.

For preparation of frozen tumour sections, freshly excised CaNT tumours were snap-frozen in liquid nitrogen. Frozen sections were cut on a cryostat at 4 μm thickness and stored at −70°C until required.

### *Immunochemical staining*

To stain for bound theophylline groups, 2 × 10$^6$ fixed cells were washed in PBS, and suspended in 1 ml PBS containing 0.5% Tween-20 and 0.1% normal goat serum for 15 min to reduce non-specific antibody binding sites; during optimisation of the staining protocol it was found that higher concentrations of goat serum reduced the specificity of binding of the antiserum. (The concentrations of first and second antibodies were also optimised for this application.) The cells were then centrifuged, resuspended in 0.25 ml polyclonal rabbit antiserum raised against theophylline and incubated for 1 h at 20°C. Following this incubation, the cells were washed and resuspended in 0.5 ml PBS containing 10 μl of affinity-isolated goat anti-rabbit IgG FITC conjugate and incubated for 1 h at 20°C. The cells were then washed and suspended in 2 ml PBS for analysis. For DNA staining, cells were treated with 1 mg ml$^{-1}$ RNAase and propidium iodide (15 μmol dm$^{-3}$) added.

Before immunoperoxidase staining for bound theophylline, frozen sections were air-dried and fixed in cold acetone (4°C, 10 min$^{1}$). Endogenous peroxidase was blocked by incubating samples for 15 min at room temperature in PBS containing 0.03% hydrogen peroxide and sodium azide (15 mmol dm$^{-3}$). Non-specific staining was blocked by pre-incubating sections with 0.1% normal goat serum. A 0.1% polyclonal rabbit anti-serum against theophylline was applied and the sections incubated for 1 h. After washing in PBS, a goat anti-rabbit IgG-horseradish peroxidase conjugate was applied at a dilution of 1:50 and incubated for 1 h. Sections were washed in PBS and incubated with diaminobenzidine HCl solution (2 mmol dm$^{-3}$) containing imidazole (1 mmol dm$^{-3}$) and hydrogen peroxide (0.006%) for a further 5 min to develop the insoluble peroxidase reaction product. Sections were lightly counterstained with haematoxylin before dehydrating and mounting.

### *Flow cytometry*

Analysis of cells was carried out on an Ortho Systems 50 cytofluorograph using excitation at 488 nm by a Coherent 5 W laser operating at 200 mW, and collection of fluorescent emission at 90° to the incident beam. Following passage through a dichroic lens to deflect scattered excitation wavelengths, the fluorescent emission from the FITC-conjugated antiserum was collected at wavelengths between 510 and 560 nm using a short band-pass filter and red fluorescence from propidium iodide, if present, above 620 nm. Cell doublets, clumps and debris were excluded by gating on the red fluorescence peak *vs* area histogram. Forward scattered light at the excitation wavelength was also collected and used to discriminate non-cellular debris (low scatter) in some experiments. At least 10,000 single cells were analysed from each sample. To estimate tumour hypoxic fractions, the limits of non-specific green staining had to be defined. A region was set on the green (FITC) *vs* red (propidium iodide) fluorescence histogram for stained cells from control tumours from mice that did not receive the injection of NITP so that the majority (e.g. >99%) of cells were excluded from the region because their green fluorescence was too low. The proportion of stained cells, from tumours exposed to NITP within this gated region was then taken as a measure of the hypoxic fraction. Cell cycle analysis was also carried out by setting appropriate regions according to DNA content on the same cytograms with the same region used to discriminate oxic and hypoxic cells.

### *Photomicroscopy*

Histological slides were observed using a Nikon Optiphot microscope and photographed on Kodak Ektachrome (ASA 100) daylight colour slide film.

*(d) Pharmacokinetics* Plasma and tumour concentrations of NITP were determined by HPLC. Blood samples were taken in heparin and the plasma separated by centrifugation. Tumours were removed, frozen in liquid nitrogen and stored with the plasma at −20°C prior to analysis. To 0.1 ml plasma was added 25 nmol benznidazole internal standard (Roche Products Ltd) and 2 ml methanol. Tumours were weighed into a tube containing 25 nmol benznidazole, 0.4 ml ice cold water was added, and the sample homogenised. Methanol (2 ml) was added and plasma and tumour extracts were then centrifuged and the supernatant taken to dryness. Samples were then reconstituted in 0.2 ml HPLC eluent (28% acetonitrile; 72% water) and an aliquot injected onto the HPLC (Waters 840) using a hypersil 5ODS column (12.5 cm × 4.6 mm) (Hichrom Ltd) with a Waters 441 detector using the 340 nm filter. Flow rate was 2 ml min$^{-1}$.

*(e) Physiology* Rectal temperature measurements were carried out on control and drug-treated tumour-bearing animals using a calibrated platinum thermometer. Breathing rate measurements were also carried out on tumour-bearing animals before and after drug administration using an airtight whole body plethysmograph (Travis *et al.*, 1979). Relative blood flow was assessed in tumours and gastrocnemius muscle by measuring $^{86}$RbCl uptake (Sapirstein, 1958). Animals were killed by cervical dislocation 90 s after administration of the radioactive tracer and tumours and muscle rapidly removed. The $^{86}$Rb content of the samples was measured in a Wallac 1282 gamma counter.

### Results

### In vitro *studies*

Following incubation of V79 Chinese hamster cells with NITP (0.1 mmol dm$^{-3}$), immunochemical fluorescent staining procedures for theophylline demonstrated more products of drug metabolism to be bound to cells incubated in hypoxic conditions compared with aerobic conditions (Figure 1a). Unbound parent drug is effectively removed during sample preparation and cannot be detected by HPLC in washed cells. The time course of metabolic binding of the theophylline side-chain to cells incubated under oxic and hypoxic conditions is shown in Figure 1b; a maximum hypoxic:oxic differential of fluorescent staining was obtained after 3–4 h incubation. The level of non-specific staining obtained with cells that had not been exposed to the drug was the same as that at the zero time point where exposure was brief (<1 min) and under aerobic conditions, metabolic binding increased only slightly. Exposure of cells to free theophylline (0.4 mmol dm$^{-3}$) under aerobic and hypoxic conditions had no effect on the level of non-specific staining. The hypoxic toxicity of the drug is not substantially greater than that of misonidazole (Figure 2); the concentration of NITP at which 50% survival is observed when cells are in contact with the

**Figure 1** **a**, Distribution of fluorescent staining for bound theophylline in oxic (unshaded area) and hypoxic cells (shaded area). Bars indicate the 95% population confidence limits. **b**, Time course of metabolites of 0.1 mmol dm$^{-3}$ NITP binding to V79 Chinese hamster cells at 37°C, detected by fluorescent immunohistochemistry: (●) hypoxic cells; (○) oxic cells. Each point represents the mean and standard error of three replicate determinations.

drug during a 7-day colony-forming assay under aerobic conditions is 0.7 mmol dm$^{-3}$ compared with 1.3 mmol dm$^{-3}$ for misonidazole.

The hypoxic:oxic differential in fluorescent staining is observed over a wide range of extra-cellular NITP concentrations *in vitro* (Figure 3a). When cells were incubated with NITP (0.1 mmol dm$^{-3}$) under a range of oxygen concentrations the value for half the hypoxic:oxic differential of metabolic binding was found to be 1,400 ppm (Figure 3b), which is similar to the value of 3,800 ppm for half of the oxygen radiosensitising effect, determined under identical conditions (Begg *et al.*, 1985).

### In vivo *studies*

Physiological measurements of tumour-bearing animals show that NITP, when injected at 0.5 $\mu$mol g$^{-1}$ in peanut oil (Figure 4) has only a small effect on rectal temperature and little effect on breathing rate. There was also no significant effect of the drug on tumour or muscle blood flow 50 min after injection (Table I). NITP appeared to be well tolerated and no deaths occurred in ten animals observed for 24 h following drug administration.

Bound theophylline groups could be readily detected immunochemically in mouse tumours within 45–60 min following treatment *in vivo* with NITP (Figure 5a), rising to a maximum value after 2 h. HPLC measurements of NITP in plasma show that the drug, when injected at 0.5 $\mu$mol g$^{-1}$ in peanut oil, rapidly reaches a maximum plasma concentration of 100 nmol ml$^{-1}$ (Figure 5b) which is sustained for a period of about 1 h; the plasma drug level subsequently falls with a half life of 32 min in mouse tumour and plasma. The parent drug can be detected in the tumours by HPLC before bound adducts can be detected immunologically; the delay before the appearance of bound adducts must reflect the time required for bioreductive metabolism to occur.

**Figure 2** Hypoxic toxicity of NITP to cells: (□) 0.5 mmol dm$^{-3}$; (△) 1.0 mmol dm$^{-3}$; (○) 2.0 mmol dm$^{-3}$. (●) oxic toxicity of 2 mmol dm$^{-3}$ NITP. Hypoxic toxicity survival curves for misonidazole under identical conditions: (——) 3 mmol dm$^{-3}$; (– – –) 5 mmol dm$^{-3}$ from Hodgkiss and Middleton (1985). At 0.1 mmol dm$^{-3}$ NITP was not toxic in oxic or hypoxic conditions during 8 h incubation. Standard errors are smaller than the sizes of the points plotted.

More bound theophylline groups were detected in cells from all tumours treated with NITP in air-breathing mice than in carbogen breathing mice (e.g. Figure 6). However, some high fluorescence was also observed when cells from control tumours not treated with the drug were stained for theophylline groups. This high control fluorescence resulted from two or more cells clumping together; it could be greatly reduced by staining DNA with propidium iodide and gating on the red fluorescence peak *vs* area signal to eliminate all particles except single cells (e.g. Figure 7). Single cell discrimination is achieved by this method because the width of the signal generated by a fluorescent particle in the flow cytometer is the sum of the beam width and the particle diameter. A comparison of signal area and peak separates the single cells from clumps because the increase in nuclear diameter is much greater for two or more G1 cells stuck together than occurs over the cell cycle, (Ormerod, 1989). The diploid G1 normal host cell population can be seen at about channel 15, the G1 aneuploid tumour cells at channel 30 and the G2 aneuploid cells at channel 60. There may be a small contamination of the G1 aneuploid cells with G2 diploid cells. Analysis of the histograms based only on single cells showed the levels of hypoxia in Table II; more hypoxic cells were found in tumours from animals breathing air compared with carbogen, and less staining was observed in control animals not treated with the drug. Work in progress suggests that similar levels of hypoxia occur in other CaNT tumours and that the reduction in tumour hypoxia observed when mice breathe carbogen correlates well with changes in the sensitivity of the tumours to fractionated radiation (Rojas *et al.*, 1990). The animals with Rh and SaF tumours were injected with NITP suspended in cremophor; similar levels of

**Figure 3** a, The dependence of NITP metabolites binding to cells on extracellular concentration: (●) hypoxic cells; (○) oxic cells. b, The oxygen-dependence of NITP metabolites binding to V79 Chinese hamster cells at 37°C, detected by fluorescent immunohistochemistry. Each point represents the mean and error of three independent determinations. Vertical lines indicate the values for half the hypoxic:oxic differential of metabolic binding (1) and half of the oxygen radiosensitising effect (2), determined under identical conditions (from Begg *et al.*, 1985).

**Table I** Mean relative blood flow measurements and standard errors in CaNT tumours and gastrocnemius muscle in five untreated animals and five animals treated with 0.5 $\mu$mol g$^{-1}$ NITP injected i.p. in peanut oil

| | *% injected $^{86}$Rb activity g$^{-1}$* | |
|---|---|---|
| | *Tumour* | *Muscle* |
| Control | 1.17±0.07 | 2.74±0.24 |
| NITP | 1.29±0.23 | 2.43±0.21 |

**Table II** Analysis of flow cytometric histograms of cell suspensions prepared from mouse tumours using data for single cells discriminated on the basis of DNA staining

| | | *% High fluorescence* | | |
|---|---|---|---|---|
| *Treatment* | | *Rh* | *CaNT* | *SaF* |
| Air | 21% $O_2$ | 29.1±1.9 (2) | 22.9±2.3 (9) | 18.8 (1) |
| Carbogen | 95% $O_2$ | 8.0 (1) | 3.8±1.6 (4) | 10.0 (1) |
| Control | No drug | 0.5±0.1 (2) | 1.6±0.7 (7) | 0.6 (1) |

Number in brackets represents the number of tumours contributing to each mean and standard error.

staining are seen in CaNT tumours when NITP is suspended in either peanut oil or cremophor (data not shown).

Immunoperoxidase staining of frozen sections of CaNT tumours showed that bound metabolites of NITP occurred mainly at the edges of tumour cords (Figure 8). Little immunoperoxidase staining was found in most tumour cords

**Figure 4** a, Measurements of rectal temperature in tumour-bearing animals: (○) untreated animals; (●) 0.5 $\mu$mol g$^{-1}$ NITP injected i.p. in peanut oil at t = 0 min. b, Measurements of breathing rate in tumour-bearing animals. (○) untreated animals; (●) 0.5 $\mu$mol g$^{-1}$ NITP injected i.p. in peanut oil at t = 0 min. Each point represents the mean and standard error of five separate determinations.

**Figure 5** a, Time course of NITP binding to CaNT tumour cells *in vivo*. b, (●) plasma and (○) tumour concentrations of NITP measured in the same animals. The plasma half-life (32 min) was determined over the period indicated by the solid line. Each point represents the mean and standard error of three separate determinations.

**Figure 6** Typical flow cytometric analysis of mouse CaNT tumour cells treated *in vivo* with NITP, following green fluorescent immunohistochemical staining. The vertical axes represent cell numbers. **a**, control tumour, no drug exposure; **b**, air-breathing mouse: **c**, carbogen-breathing mouse.

within 5–6 cell diameters of the central blood vessels, or in the necrotic material surrounding the tumour cords. Occasionally, however, a tumour cord was found in which immunoperoxidase staining occurred adjacent to the blood vessel and throughout that particular cord (Figure 8, lower right). Such heavily stained tumour cords may represent regions that became acutely hypoxic by blood-vessel occlusion at a time when the probe had already diffused into the tumour.

### Discussion

Numerous studies have demonstrated the hypoxia-specific binding of isotopically labelled 2-nitroimidazoles to cellular components and it has been possible to develop an immunohistochemical method for detecting bound metabolites of a fluorinated nitroimidazole (Raleigh *et al.*, 1987). We have used a commercial antiserum to theophylline to quantify similarly bound metabolites of a 2-nitroimidazole with a theophylline side-chain. *In vitro* studies have shown bioreductive binding of NITP to be highly hypoxia-specific and to have a similar oxygen-dependence to radiosensitivity. While there may be a small overlap between the range of intensities of fluorescent staining seen with pure oxic and hypoxic cell populations, the mean fluorescence of the two groups is well separated. Metabolic binding of NITP to hypoxic cells can be readily demonstrated under conditions where there is no toxicity. With long incubation times, a plateau is observed in the amount of bound drug detectable in cells, although the cells are still completely viable. We have observed similar binding curves with other nitro-aromatic compounds tested as fluorescent probes for hypoxic cells, under identical conditions (e.g. Begg *et al.*, 1985); the apparent plateau in drug binding may reflect further metabolism of bound adducts to non-recognisable forms.

The drug is tolerated well *in vivo* with little effect observed on body temperature, breathing rate or relative tumour and

**Figure 7** Typical flow cytometric contour plots of DNA staining *vs* green fluorescent immunohistochemical staining for theophylline of mouse CaNT tumour cells treated *in vivo* with NITP. **a**, control tumour, no drug exposure: **b**, air-breathing mouse: **c**, carbogen-breathing mouse. The regions used to generate the high green fluorescence data in Table II are indicated. The inset shows the DNA distribution as a histogram of cell number *vs* DNA content for this tumour.

**Figure 8** Immunoperoxidase staining for bound theophylline of frozen sections of the CaNT mouse mammary carcinoma treated *in vivo* with NITP. The bar represent 0.2 mm.

**Figure 9** The structure of 7-(4'-(2-nitroimidazol-l-yl)-butyl)-theophylline (NITP).

muscle blood flow. The maximum plasma levels obtained *in vivo* following administration of NITP (0.5 $\mu$mol $g^{-1}$) in peanut oil are well below the concentrations at which any hypoxic or oxic toxicity can be detected *in vitro*. The long plateau observed in the concentration of NITP in the plasma suggests that this method of drug administration may result in slow sustained release of the drug, thereby achieving a significant tumour concentration for sufficient time for bioreductive metabolism to occur. The extracellular NITP concentration-dependence of hypoxic-oxic differential staining observed *in vitro* suggests that it may be possible to use smaller amounts of NITP *in vivo*, although in practice it has been found that reducing the amount of NITP administered reduces the differential between the highest levels of staining and the non-specific background staining.

Bound adducts can also be readily demonstrated in cells from all tumours treated *in vivo* with this compound. Furthermore, the large reduction in binding observed when the animals breathe carbogen suggests that we are observing a real cell population where the oxygen supply is normally restricted. A similar reduction in tumour hypoxia when mice, adapted to breathing 10% oxygen, breathe carbogen has been shown to reduce the amount of labelled misonidazole binding to tumour cells (Franko, 1986). Radiobiological studies have estimated mean hypoxic fractions of 30% (95% confidence interval 14–62%) for the Rh tumour, 38% (95% confidence interval 26–55%) for the CaNT tumour and 69% (95% confidence interval 42–100%) for the SaF tumour in animals breathing air (Moulder & Rockwell, 1984). While similar hypoxic fractions to the radiobiological mean values are derived here from the flow cytometry data (Table II), a direct comparison cannot be made as the estimates of radiobiological hypoxia are not contemporary and some changes in tumour growth characteristics have been noted since these estimates were made (e.g. Anderson *et al.*, 1989). Although similar hypoxic fractions have been estimated from measurements of xanthine oxidase and xanthine dehydrogenase (Anderson *et al.*, 1989), these estimates also require radiobiological calibration.

Experiments carried out *in vitro* suggest that NITP will slightly underestimate the degree of hypoxia, compared with the radiobiological effects, so that those cells that are detected are likely to be sufficiently deprived of oxygen to be radioresistant. The precision with which NITP detects small proportions of cells with intermediate levels of oxygen is limited by the non-specific background staining, but the mean fluorescence of completely hypoxic cells should be well separated from that of completely oxic cells. In practice the continuous distribution of oxygen tensions expected within tumours results in a continuous distribution of staining intensities, making fine distinctions of oxygen levels difficult. We have shown that the changes in hypoxic fraction measured using NITP correlate well with changes in tumour radiosensitivity in animals breathing air, carbogen or oxygen at the time of treatment (Rojas *et al.*, 1990).

It has been shown that much of the bioreductive binding of misonidazole is to acid-soluble components of the cell such as glutathione (Miller *et al.*, 1982; Smith & Born, 1984); if NITP metabolites behaved similarly, fixation and permeablisation of the cells would presumably extract such low molecular weight conjugates. However, the immunochemical procedures employed here are sensitive enough to detect the remaining adducts to high molecular weight components of the cell. Although it has been reported that 90% (Miller *et al.*, 1982) and 41% (Smith, 1984; Smith & Born, 1984) of the total $^{14}C$ and $^{3}H$-labelled misonidazole binding to the macromolecular fraction of cells is associated with the RNA, 80% of the staining for bound adducts of NITP is obtained in cells treated with RNAase to enable specific staining of the DNA with propidium iodide. The improved flow cytometric analysis of the cell suspension (based on discrimination of single cells) that can be carried out following staining of the DNA compensates for the small reduction in the amount of staining for the hypoxia marker resulting from the removal of RNA. Almost all of the highly fluorescent particles found when cell suspensions from tumours which have not been exposed to NITP are stained for bound theophylline are non-cellular debris or clumps of one or more cells, and can be identified and excluded from analysis on the basis of DNA content. DNA staining also allows the distribution of hypoxic cells throughout the cell cycle to be studied; preliminary data suggest that hypoxia is found throughout the cell cycle among the aneuploid tumour cells, but that lower levels of hypoxia may be found in the G1 diploid normal host cells. Alternative second staining procedures may also allow tumour hypoxia to be correlated with other parameters such as labelling index, markers for specific cell types and thiol status.

In frozen sections of the CaNT tumour stained for the presence of bound probe metabolites, immunoperoxidase staining is found in the histologically intact cells at the periphery of tumour cords where oxygen tensions would be expected to be low (Gray *et al.*, 1953; Thomlinson & Gray, 1955). As necrotic regions of the tumour are not markedly stained, the flow cytometric measurements of tumour hypoxia exclude the necrotic material which is unlikely to influence the efficacy of treatment. Similar distributions of staining were observed by Raleigh *et al.* (1987) in the Walker carcinoma using immunofluorescent detection of metabolites of a fluorinated nitroimidazole, although their stain appears to stain tumour necrosis as well as several layers of healthy cells adjacent to the necrotic regions. Occasionally tumour cords are seen where the immunoperoxidase staining is adjacent to the blood vessels. This may represent an example of acute hypoxia where the blood flow has become temporarily disrupted at a time when the probe was available in the tumour cells for bioreductive metabolism.

Fluorescent immunochemical staining of bound metabolites of NITP in cell suspensions, followed by flow cytometric analysis provides a rapid, convenient assay for the hypoxic fraction in solid tumours *in vivo* with considerable potential in the investigation of experimental radiotherapy in animal models (Rojas *et al.*, 1990). While further development work is still required, these early results illustrate that immunochemical detection of bound metabolites of suitable nitroaromatic compounds could provide the basis for a simple test to estimate the hypoxic fraction in clinical tumour samples.

We thank Dr S. Hill and Mrs K. Wilson for assistance with blood flow measurements and for providing tumour-bearing animals and Dr N.J. McNally and Dr P. Wardman for helpful discussion. This work is supported by the Cancer Research Campaign.

**References**

ANDERSON, R.F., PATEL, K.B., REGHEBI, K. & HILL, S.A. (1989). Conversion of xanthine dehydrogenase to xanthine oxidase as a possible marker for hypoxia in tumours and normal tissues. *Br. J. Cancer*, **60**, 193.

BEGG, A.C., HODGKISS, R.J., MCNALLY, N.J., MIDDLETON, R.W., STRATFORD, M.R.L. & TERRY, N.H.A. (1985). Fluorescent markers for hypoxic cells: a comparison of two compounds on three cell lines. *Br. J. Radiol.*, **58**, 645.

BUSH, R.S., JENKIN, R.D.T., ALLT, W.E.C. & 4 others (1978). Definitive evidence for hypoxic cells influencing cure in cancer therapy. *Br. J. Cancer*, **37** Suppl III, 302.

CHAPMAN, J.D., FRANKO, A.J. & SHARPLIN, J. (1981). A marker for hypoxic cells in tumours with potential clinical applicability. *Br. J. Cancer*, **43**, 546.

CHURCHILL-DAVIDSON, I., FOSTER, C.A., WIERNIK, G. & 4 others (1966). The place of oxygen in radiotherapy. *Br. J. Radiol.*, **39**, 321.

DISCHE, S. (1985). Chemical sensitizers for hypoxic cells: a decade of experience in clinical radiotherapy. *Radiotherapy and Oncol.*, **3**, 97.

DISCHE, S., SAUNDERS, M.I. & WARBURTON, M.F. (1986). Hemoglobin, radiation, morbidity and survival. *Int. J. Radiat. Oncology Biol. Phys.*, **12**, 1335.

FRANKO, A.J. (1986). Misonidazole and other hypoxia markers: metabolism and applications. *Int. J. Radiat. Oncology Biol. Phys.*, **12**, 1195.

FRANKO, A.J. & CHAPMAN, J.D. (1982). Binding of $^{14}C$-misonidazole to hypoxic cells in V79 spheroids. *Br. J. Cancer*, **45**, 694.

GARRECHT, B.M. & CHAPMAN, J.D. (1983). The labelling of EMT-6 tumours in BALB/C mice with $^{14}C$-misonidazole. *Br. J. Cancer*, **56**, 745.

GRAY, L.H., CONGER, A.D., EBERT, M., HORNSEY, S. & SCOTT, O.C.A. (1953). Concentration of oxygen dissolved in tissues at time of irradiation as a factor in radiotherapy. *Br. J. Radiol.*, **26**, 638.

HILL, S.A., SMITH, A. & DENEKAMP, J.D. (1989). Reduced thermal sensitivity of the vasculature in a slowing growing tumour. *Int. J. Hypertherm.*, **5**, 359.

HODGKISS, R.J. & MIDDLETON, R.W. (1985). Effects of glutathione depletion using buthionine sulphoximine on the cytotoxicity of nitroaromatic compounds in mammalian cells in vitro. *Biochem. Pharm.*, **34**, 2175.

HODGKISS, R.J., MIDDLETON, R.W., STRATFORD, M.R.L. & DEL BUONO, R. (1987). Toxicity of 3-nitrosaphthalimides to V79 379A Chinese hamster cells. *Biochem. Pharm.*, **36**, 1483.

MILLER, G.G., NGAN-LEE, J. & CHAPMAN, J.D. (1982). Intracellular localization of radioactively labelled misonidazole in EMT-6 tumour cells in vitro. *Int. J. Radiat. Oncol. Biol. Phys.*, **8**, 741.

MOULDER, J.E. & ROCKWELL, S. (1984). Hypoxic fractions of solid tumours: experimental techniques, methods of analysis, and a survey of existing data. *Int. J. Radiat. Oncol. Biol. Phys.*, **10**, 695.

MUELLER-KLIESER, W., VAUPEL, P., MANZ, R. & SCHMIDSEDER, R. (1981). Intracapillary oxyhemoglobin saturation of malignant tumours in humans. *Int. J. Radiat. Oncol. Biol. Phys.*, **7**, 1397.

ORMEROD, M.G. (1990). Analysis of DNA – general methods. *In Flow Cytometry: a Practical Approach*, Ormerod, M.G. (ed.) IRL Press: Oxford.

OVERGAARD, J., HANSEN, H.S., JORGENSEN, K. & HJELM-HANSEN, M. (1986). Primary radiotherapy of larynx and pharynx carcinoma – an analysis of some factors influencing local control and survival. *Int. J. Radiat. Oncol. Biol. Phys.*, **12**, 515.

RALEIGH, J.A., FRANKO, A.J., KOCH, C.J. & BORN, J.L. (1985). Binding of misonidazole to hypoxic cells in monolayer and spheroid culture: evidence that a side-chain label is bound as efficiently as a ring label. *Br. J. Cancer*, **51**, 229.

RALEIGH, J.A., FRANKO, A.J., TREIBER, E.O., LUNT, J.A. & ALLEN, P.S. (1986). Covalent binding of a fluorinated 2-nitroimidazole to EMT-6 tumours in Balb/C mice: detection by F-19 nuclear magnetic resonance at 2.35 T. *Int. J. Radiat. Oncol. Biol. Phys.*, **12**, 1243.

RALEIGH, J.A., MILLER, G.G., FRANKO, A.J., KOCH, C.J., FUCIARELLI, A.F. & KELLEY, D.A. (1987). Fluorescence immunohistochemical detection of hypoxic cells in spheroids and tumours. *Br. J. Cancer*, **56**, 395.

RASEY, J.S., KROHN, K.A., GRUNBAUM, Z., CONROY, P.J., BAUER, K. & SUTHERLAND, R.M. (1985). Further characterisation of 4-bromomisonidazole as a potential detector of hypoxic cells. *Radiat. Res.*, **102**, 76.

ROJAS, A., HODGKISS, R.J., CARL, U, G.D., PARRICK, J. & COLLIER, J.M. (1990). Enhancement of tumour radiosensitivity and reduced hypoxia-dependent binding of a 2-nitroimidazole with normobaric oxygen and carbogen. *Submitted to Radiotherapy and Oncology*.

SAPIRSTEIN, L.A. (1958). Regional blood flow by fractional distribution of indicators. *Am. J. Physiol.*, **193**, 161.

SMITH, B.R. (1984). Hypoxia-enhanced reduction and covalent binding of [2-$^{3}$H]misonidazole in the perfused rat liver. *Biochem. Pharmacol.*, **33**, 1379.

SMITH, B.R. & BORN, J.L. (1984). Metabolism and excretion of [$^{3}$H] misonidazole by hypoxic rat liver. *Int. J. Radiat. Oncol. Biol. Phys.*, **10**, 1365.

THOMLINSON, R.H. & GRAY, L.H. (1955). The histological structure of some human lung cancers and the possible implications for radiotherapy. *Br. J. Cancer*, **9**, 539.

TRAVIS, E.L., VOJNOVIC, B., DAVIES, E.E. & HIRST, D.G. (1979). A plethysmographic method for measuring function in locally irradiated mouse lung. *Br. J. Radiol.*, **52**, 67.

URTASUN, R.C., CHAPMAN, J.D., RALEIGH, J.A., FRANKO, A.J. & KOCH, C.J. (1986). Binding of $^{3}$H-misonidazole to solid human tumours as a measure of tumour hypoxia. *Int. J. Radiat. Oncol. Biol. Phys.*, **12**, 1263.